# EEG machine learning for accurate detection of cholinergic intervention and Alzheimer’s disease

**DOI:** 10.1038/s41598-017-06165-4

**Published:** 2017-07-18

**Authors:** Sonja Simpraga, Ricardo Alvarez-Jimenez, Huibert D. Mansvelder, Joop M. A. van Gerven, Geert Jan Groeneveld, Simon-Shlomo Poil, Klaus Linkenkaer-Hansen

**Affiliations:** 1 Department of Integrative Neurophysiology, CNCR, Neuroscience Campus Amsterdam, Vrije Universiteit Amsterdam, Amsterdam, The Netherlands; 2 Centre for Human Drug Research, Leiden, The Netherlands; 3 Department of Neurology, VU University Medical Center, Amsterdam, The Netherlands; 4 NBT Analytics BV, Amsterdam, The Netherlands

## Abstract

Monitoring effects of disease or therapeutic intervention on brain function is increasingly important for clinical trials, albeit hampered by inter-individual variability and subtle effects. Here, we apply complementary biomarker algorithms to electroencephalography (EEG) recordings to capture the brain’s multi-faceted signature of disease or pharmacological intervention and use machine learning to improve classification performance. Using data from healthy subjects receiving scopolamine we developed an index of the muscarinic acetylcholine receptor antagonist (mAChR) consisting of 14 EEG biomarkers. This mAChR index yielded higher classification performance than any single EEG biomarker with cross-validated accuracy, sensitivity, specificity and precision ranging from 88–92%. The mAChR index also discriminated healthy elderly from patients with Alzheimer’s disease (AD); however, an index optimized for AD pathophysiology provided a better classification. We conclude that integrating multiple EEG biomarkers can enhance the accuracy of identifying disease or drug interventions, which is essential for clinical trials.

## Introduction

An increasing number of drug candidates are being tested for their ability to modify disease or alleviate symptoms of brain disorders^1^; however, to test these new pharmacological interventions and improve monitoring of the therapeutic response, informative and robust endpoints are urgently needed^2–4^. Clinical trials in central nervous system (CNS) drug development focus on behavioral and cognitive performance outcome measures of drug efficacy; however, quantitative electroencephalography (EEG) is gaining recognition in the field as a source of surrogate endpoints in early-phase studies^5^. EEG offers insight into the mode of action of the pharmacological intervention, because of the high temporal resolution of electrophysiological measures^6, 7^. Still, it remains an important challenge to advance EEG biomarker analysis for enhanced prediction of therapeutic effects in clinical trials.

Scopolamine is the most extensively used pharmacological model of cognitive impairment^8^. As a selective competitive muscarinic receptor (mAChR) antagonist, it induces temporary deficits in cognitive functions that depend on the cholinergic system^9^. Scopolamine has a high affinity for all five muscarinic receptor subtypes (M1–M5) and a negligible affinity for histaminergic and dopaminergic receptors^10^. Muscarinic receptors are widely present in brain areas involved in attention and memory, and intravenous administration of scopolamine indeed causes impairments to these brain functions^11, 12^. The scopolamine challenge test has been used in drug development to demonstrate the pharmacological activity of putatively cognition-enhancing compounds by reversal of scopolamine-induced cognitive deficits in healthy volunteers^13–20^.

EEG biomarkers have the potential to objectively determine whether reversal of scopolamine effects by a cholinergic compound is successful. In humans, scopolamine administration increases the power of delta and theta activity, while alpha- and beta-frequency activity is reduced^9, 12, 21^. It has been hypothesized that deficits of cholinergic signaling contribute to the EEG slowing in Alzheimer’s disease^22, 23^, which is also supported by the reversal of EEG slowing by cholinergic drugs^24, 25^. Unfortunately, current biomarkers lack the desired accuracy for monitoring disease status or therapeutic response, because of large inter-individual variability compared to the often subtle drug-related changes. Most commonly, the functional state of the brain is assessed merely using one type of biomarker^26–29^; however, pathophysiology is often expressed as changes to multiple properties of neuronal oscillations. Consequently, different biomarker algorithms may quantify distinct aspects of the brain’s functional state. Combining these may increase the accuracy of disease diagnosis and assessment of drug interventions^30–34^. Here, we use machine learning to show that the complementary information of different EEG biomarkers can indeed be combined into an accurate index for better decision-making in clinical trials.

For this purpose, data from four clinical trials were used. In Trials 1 and 2 healthy adults received a cognitive impairment challenge test using the muscarinic anticholinergic drug scopolamine. From these two trials combined we derived the mAChR index. In Trial 3 healthy elderly subjects received the same scopolamine challenge and Trial 4 consisted of patients with mild to moderate AD with no intervention. From Trials 3 and 4 we derived an AD index, which we contrasted with the mAChR index to investigate how the scopolamine challenge captures the cholinergic dysfunction that occurs in AD.

## Results

### Scopolamine affects both spectral and temporal dynamics of the EEG

**Figure 1:**
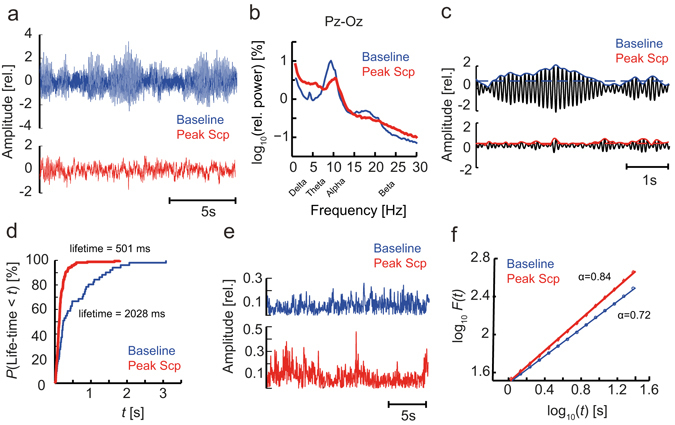
Spectral and temporal correlation biomarkers exhibit sensitivity to scopolamine administration. (**a**) EEG of a subject in the baseline (*blue*) and scopolamine (*red*) condition. (**b**) Grand average normalized power spectra indicate large effects of scopolamine, most notably a reduction of power in the alpha and beta bands, and an increase of delta and theta power. (**c**) Oscillation dynamics were studied by extracting the amplitude envelope from band-pass filtered data (e.g., the alpha band, *black*) using the Hilbert transform (*blue*, *red*) and a median-amplitude threshold to determine the onset and offset of a burst. (**d**) A cumulative probability distribution of all oscillation bursts revealed a tendency towards longer alpha bursts in the baseline condition. (**e**) Amplitude envelopes of beta oscillations (13–30 Hz) suggest a more complex temporal structure in the peak scopolamine (*red*) than in the baseline (*blue*) condition on time scales of seconds to tens of seconds. (**f**) The long-time scale differences in beta oscillations are reflected in the grand average DFA showing larger scaling exponents for peak scopolamine (*red*) than for the baseline recording (*blue*). All figures were based on the Pz-Oz channel.

To gain a comprehensive understanding of the effects of scopolamine on the EEG, we employed biomarker algorithms characterizing spectral content as well as temporal dynamics of neuronal oscillations. The spectral content was estimated using power spectrum analysis of the broadband EEG signals (Fig. 1a,b). The short-time scale temporal structure of narrow-band oscillations was quantified by extracting the amplitude envelope and applying oscillation-bursts lifetime analysis (Fig. 1c,d), whereas temporal dynamics on longer time scales was quantified using detrended fluctuation analysis (Fig. 1e,f).

**Figure 2:**
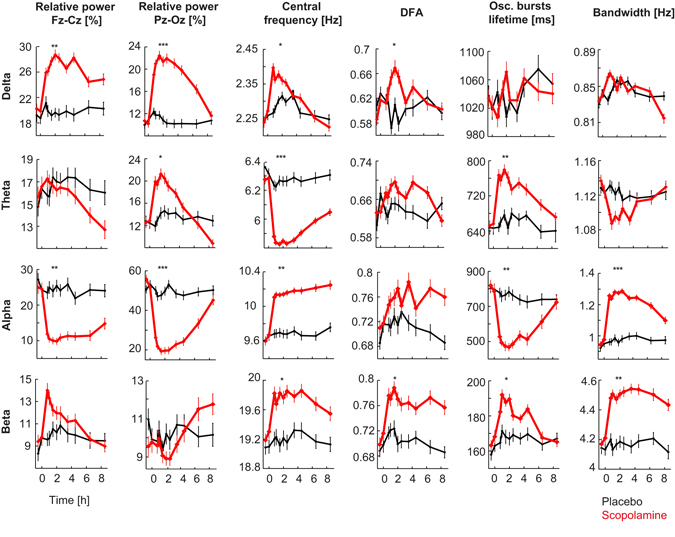
Scopolamine affects many characteristics of the EEG. Time dependence of different EEG biomarkers (columns) and frequency bands (rows) for placebo (*black*) and scopolamine (*red*). All biomarkers are shown as averages over the 2 channels, except for relative power, for which the Fz-Cz and Pz-Oz channels are shown separately, because the effects in the delta and beta bands were opposite for the two derivatives. Sixteen biomarkers were significantly affected by scopolamine, with the peak effect occurring 1.5 h after administration. The values plotted are group means and standard errors of the mean computed for the within-subject design^67^. Significance levels: * denotes *p* < 0.05, **p < 10^−5^, ***p < 10^−10^; Bonferroni corrected for multiple comparisons.

To examine the effects of scopolamine administration compared to placebo, we quantified these differences systematically at 11 time points from 30 minutes before to 8.5 hours after scopolamine and placebo administration. In Fig. 2, we display the results as time-dependent biomarker curves of relative power, central frequency, bandwidth, oscillation-burst lifetime, and DFA in the columns and the frequency bands in the rows. A significant effect of scopolamine compared to placebo was observed for several biomarkers (Wilcoxon rank sum test at 1.5 h after administration, Bonferroni corrected). Despite all of these robust effects, if biomarkers carry complementary information about scopolamine-induced EEG changes, then it may be possible to combine this information into a more sensitive measure of the anticholinergic effect compared to using any of the individual biomarkers.

### Integrating biomarkers enhances classification

We used machine learning techniques to find the biomarkers that best distinguish the baseline from the peak scopolamine condition. In order to do this, we performed classification on the baseline recording and the EEG recorded 1.5 h after administration of scopolamine (cf., Fig. 2). The baseline was used as opposed to the placebo condition to eliminate variation between days.

**Figure 3:**
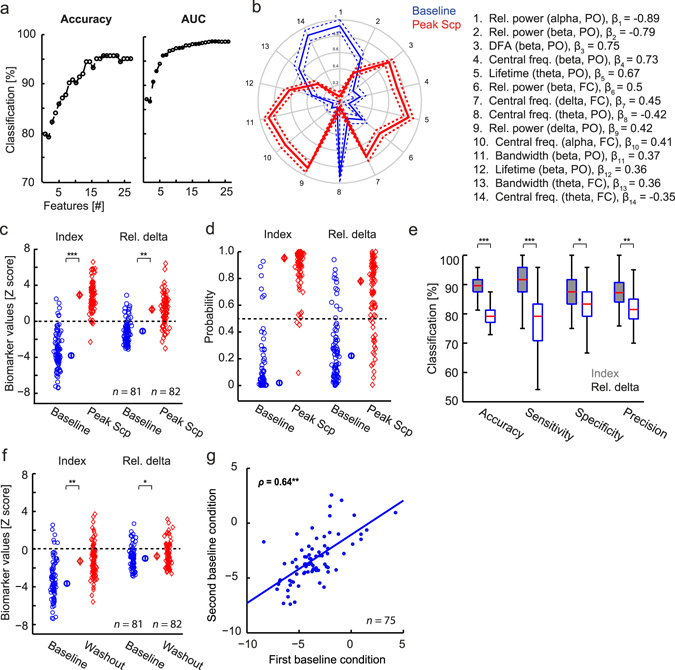
Enhanced detection of scopolamine-induced EEG changes using machine learning. (**a**) Classification performance increased with the number of features included in the integrated index. (**b**) All of the biomarkers selected by elastic net logistic regression for inclusion in the mAChR index differed significantly between baseline and peak scopolamine. Biomarkers are ordered by their absolute weights, decreasing clockwise from the top. Weights (*β*) are listed next to each biomarker in the legend (PO denotes Pz-Oz and FC denotes Fz-Cz). The values plotted on the spider plot are the z-score group means and standard error of the mean, normalized to [0, 1] by subtracting the minimum across all biomarkers and dividing with the largest range present (i.e., the difference between the minimum and maximum value found for the biomarkers with the largest difference). (**c**) The mAChR index was more sensitive to the scopolamine (Scp) intervention than relative delta power. The plot shows z-scored biomarker values per subject recording. Singled-out symbols represent median values per group with standard error bars. The dashed line indicates the threshold of the classifier to predict the recordings as a baseline (below) or a peak scopolamine (above) recording. (**d**) Same as (c) but instead of z-scored biomarker values, predictive probabilities obtained from the classifier are shown. (**e**) Classification performance of baseline vs scopolamine at the peak drug effect using 100 cross-validations is significantly higher for the mAChR index (grey boxplots) than the relative delta power (white boxplots). (**f**) The superiority of the mAChR index was also pronounced at washout. Relative delta power in the scopolamine condition was almost back to normal at 8.5 h after administration, whereas the mAChR index produced a highly significant effect. (**g**) The mAChR index has high test-retest reliability across weeks. Significance levels legend for this figure: * denotes *p* < 0.05, **p < 10^−10^, ***p < 10^−20^, using Wilcoxon rank sum test.

An initial integrated index was developed using elastic net on the data from healthy subjects (*n* = 83 males, Trial 1 and 2, see Methods) that received scopolamine, while allowing a fraction—determined by the algorithm—of the 40 available biomarkers to be included. Subsequently, to simplify the composition of the index, biomarkers with non-zero weights were sorted by decreasing absolute weight and added incrementally in that order (i.e., starting from an empty set, we added the biomarker with the largest absolute weight etc.) to evaluate the gain of including each subsequent biomarker to the classifier. Accuracy and area under curve increased with the number of features included in the index up until a maximum performance was reached (Fig. 3a). We defined the optimal index to be the one with the smallest number of features for which the average of all performance measures had saturated. We estimated this number to be 14 (Fig. 3a) according to the “elbow” method^35^; together, this set of 14 biomarkers and their associated weights make up the integrated mAChR index (Fig. 3b).

The mAChR index had excellent performance when training and testing on the same data (accuracy 95%, sensitivity 96%, specificity 93%, precision 93% and area under curve 0.98), and much higher than the single-best biomarker (accuracy 82%, sensitivity 80%, specificity 84%, precision 83% and area under curve 0.87), which was relative delta power (Fig. 3c,d). Accordingly, the difference between the baseline predicted group and the peak scopolamine predicted group (Fig. 3c) was much more pronounced for the mAChR index (*p* = 2 * 10^−26^, Wilcoxon rank sum test) than for relative delta (*p* = 6 * 10^−16^). The single-best biomarker was determined by performing elastic net classification using each of the features alone and then ranking them by the average of the different classification outcome measures. To obtain a more accurate estimate of the classification performance, we used cross-validation. The difference in performance per cross-validation was due to different subsets of subjects used for training and testing in each iteration, resulting in slightly different biomarker selections and weights. The number of iterations we performed was 100, because performance outcome values remained stable when using ≥100 cross-validations. Cross-validation on these two datasets (Fig. 3e) resulted in an accuracy of 90 ± 2%, sensitivity of 92 ± 4%, specificity of 88 ± 4% and precision of 88 ± 3%, which is still very high and significantly higher than using just relative delta: accuracy of 79 ± 2%, sensitivity of 79 ± 4%, specificity of 83 ± 4% and precision of 81 ± 3% (*p* = 9 * 10^−29^ for accuracy, Wilcoxon rank sum test). Interestingly, the difference between the baseline predicted and scopolamine predicted groups was also more significant for the mAChR index (*p* = 9 * 10^−10^, Wilcoxon rank sum test) than for relative delta (*p* = 0.02) when tested at washout—8.5 h after scopolamine administration (Fig. 3f).

### The mAChR index is robust and generalizable

**Figure 4:**
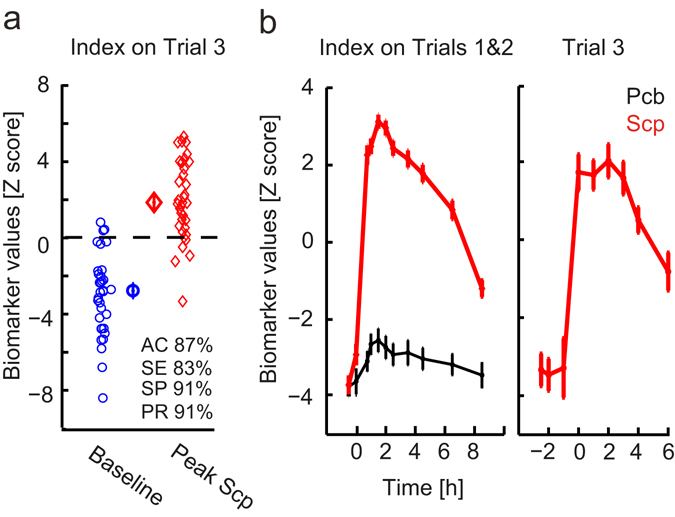
The mAChR index generalizes to a new cohort of subjects. (**a**) The mAChR index also generalizes to a cohort of healthy elderly subjects receiving the same scopolamine intervention (Trial 3), with validation accuracy 87%, sensitivity 83%, specificity 91% and precision 91%. (**b**) Time dependence curves demonstrate the generalizability of the index at all the time points. The mAChR index for placebo (*black*) and scopolamine (*red*) is shown for Trials 1&2 used for developing the index and for independent data from Trial 3 (in the latter there was no placebo condition). The values plotted are group means and standard errors of the mean computed for the within-subject design^67^.

Test-retest stability is an important quality of a biomarker. We, therefore, compared the mAChR index scores of baseline recordings from two separate days in 75 subjects (Trial 1 and 2) and observed a strong correlation of 0.64 (Spearman correlation, *p* = 2.5 * 10^−10^, Fig. 3g). To further demonstrate the generalizability of the mAChR index, we applied it to an independent cohort of healthy elderly subjects (Trial 3, see Methods) receiving a similar scopolamine intervention. Interestingly, in spite of the difference between the age groups in Trials 1–2 and 3, we observed an index performance very close to the cross-validation on the adult cohort (Fig. 4a; accuracy 87%, sensitivity 83%, specificity 91% and precision 91%). Importantly, the index also generalized to the other measurement time points both for Trials 1, 2 and 3 (Fig. 4b). Applying the mAChR index to Trial 3 was an independent validation because the index was obtained solely from Trial 1 and 2 data and applied to Trial 3 without retraining (i.e. with identical weights).

### Scopolamine is a valid model of AD pathophysiology

**Figure 5:**
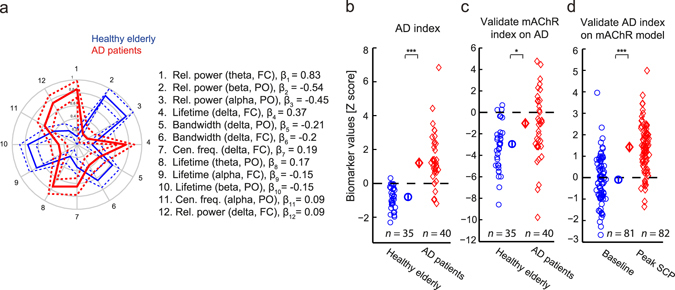
The Alzheimer’s index captures scopolamine-induced effects and validates scopolamine as a model of AD pathophysiology. (**a**) Illustration of the twelve biomarkers composing the AD index. Several of these biomarkers also compose the scopolamine index, with the same directionality of change in both the scopolamine-induced cognitive impairment and Alzheimer’s disease. The values plotted are as explained in Fig. 3B. (**b**) AD index separates healthy elderly from Alzheimer’s disease patients with high precision. (**c**) The mAChR index discriminates the healthy elderly and AD patients albeit less accurately than the AD index. (**d**) Validating the AD index on the scopolamine data gives much better discrimination.

To test the validity of scopolamine as a model for the cholinergic dysfunction that occurs in AD, we next developed an AD index. Using the patients with mild to moderate AD (Trial 4) and the age-matched healthy elderly subjects (Trial 3), we derived an AD index consisting of 12 biomarkers (Fig. 5a), of which 5 are the same as those in the mAChR index. The AD index performed with an accuracy of 92%, sensitivity 87%, specificity 97% and precision 97% when training and testing on the same data (Fig. 5b). Cross-validated, the respective performances were 73 ± 6%, 73 ± 9%, 70 ± 10% and 75 ± 7%. Next, we investigated the relation of the AD index to the mAChR index, comparing their abilities to discriminate healthy elderly subjects from patients with mild to moderate AD, or discriminate baseline from peak scopolamine. Applying the mAChR index on healthy elderly and AD patients, we observed that it was able to discriminate them, with an accuracy of 62%, sensitivity 35%, specificity 91% and precision 81% (Fig. 5c). The separation was better when applying the AD index on subjects before and after scopolamine intervention, with accuracy 72%, sensitivity 89%, specificity 54% and precision 66%. It should be noted that feature selection and model training of the mAChR index were performed exclusively on data from Trial 1 and 2 and then tested on Trial 3 and 4 without retraining, and vice versa. Taken together our results show that the mAChR index captures cholinergic dysfunction that occurs in AD. This is reflected by several biomarkers shared between the mAChR and the AD index, as well as the mutual ability to distinguish subjects with AD or subjects given scopolamine, respectively. Also, the good performance of the AD index in discriminating healthy elderly from AD patients further demonstrates the value of multi-biomarker classification schemes.

## Discussion

Resting-state EEG signals are complex and information rich^36^. A variety of spectral, spatial and temporal biomarker algorithms have been used to uncover brain electrophysiological changes in disease or with pharmacological intervention^30, 37, 38^; however, they all have too low sensitivity and specificity to become standard tools in hospitals and clinical trials^5, 39^. To address this problem, we tested whether extensive characterization of EEG using multiple biomarkers and subsequent application of machine learning could improve the accuracy of classifying disease state or drug intervention. We developed a mAChR index with superior sensitivity and specificity to the complex structure of the EEG changes induced by scopolamine intervention compared to any single biomarker. The enhanced accuracy could be of great value in evaluating the efficacy of drugs that aim to induce effects opposite to scopolamine, e.g., for the treatment of AD and schizophrenia-related cognitive impairment. We believe our methodological approach could prove invaluable in a wide range of challenge tests used in CNS drug development.

Scopolamine is known to decrease alpha power and increase relative delta and theta power, mainly in posterior regions^12, 40, 41^. These EEG changes are hallmarks of cognitive impairment associated with AD^24, 42, 43^ and also observed in our healthy subjects after scopolamine administration (Fig. 2). Other biomarkers affected by scopolamine included the oscillation burst lifetime duration biomarker, which decreased in the alpha band (Fig. 1a,c,d) and increased in the theta band (Fig. 2) as observed also in early-stage AD^30^. Scopolamine produced an increase in DFA in all frequency bands, albeit this effect was only sufficiently strong in the beta band for inclusion in the mAChR index (Figs 1e,f and 2). The mAChR index also comprised the central frequency effects of a decrease in the theta band and an increase in the alpha and beta bands (Fig. 2). Changes in the central frequency and bandwidth were correlated, decreasing in the theta band and increasing in alpha and beta bands. Larger bandwidth could be associated with less frequency stability of alpha and beta oscillations, which has previously been linked to a less efficient working memory^44^.

A similar analysis using machine learning for classifying scopolamine effects on the EEG has been developed in the past^45^; however, with different features and analysis methods used and without reporting the classification performance of the index; therefore, it cannot readily be compared with our findings. Our cross-validation (training and testing on different data) resulted in a remarkable performance (Fig. 3c–e). Importantly, the test-retest reliability of the mAChR index across two baseline recordings was very high (Fig. 3g), and validation on an independent set of data confirmed that the index generalizes to new cohorts (Fig. 4a,b). Interestingly, when classifying on the washout period, classification with the mAChR index was highly significant, whereas that of the single-best biomarker was only marginally significant (Fig. 3f), suggesting that clinical situations with many more subtle drug effects will gain substantially from the proposed machine learning and data-integration approach. This is particularly useful in view of the fact that acute pharmacodynamics effects of pro-cognitive, cholinergic compounds are often difficult to measure in healthy subjects or patients with AD^46–49^.

To examine the validity of scopolamine as a model of AD pathophysiology, we applied the mAChR index to healthy elderly controls and patients with AD. We also derived an AD index to test whether scopolamine-induced EEG changes resemble those of AD. Applying the mAChR index to AD patients and controls we observed that it indeed showed an effect (Fig. 5c); however, it discriminated less accurately than the AD index and with a shift in the classification threshold. According to the mAChR index, some AD patients were misclassified as healthy elderly, presumably because the EEG is affected more strongly by scopolamine than mild (or moderate) AD. Nonetheless, because the differences were in the same direction, applying the AD index to the subjects on scopolamine resulted in a much better separation (Fig. 5d). The misclassification here was opposite: a few baseline recordings were predicted as scopolamine, because the differences between healthy elderly and AD are weaker and, therefore, the separation threshold for the AD index is lower. It is interesting to note the similarity between the effect of applying the mAChR index to AD patients and controls (Fig. 5c) and applying the index on the scopolamine washout period (Fig. 3f). Together, this suggests that scopolamine is a good cognitive impairment model for AD, mimicking the changes seen in AD patients; however, with a difference in the magnitude of effects. Nicotinic blockade added to the muscarinic anticholinergic effects might better resemble changes reflected in both indices and might further explain the difference observed between AD and scopolamine peak effects^50–53^.

Integrating information from multiple EEG biomarkers has an advantage over the standard power spectrum, because of the often subtle changes from baseline and the considerable inter-individual variability at baseline for EEG and cognitive tests. This approach also reduces the multiple-comparisons problem when analyzing several EEG biomarkers in clinical trials. A specific mAChR index may help to quantify effects of pro-cognitive cholinergic compounds, and muscarinic agonists in particular. Reversal of detrimental effects induced by scopolamine on cognitive performance has been demonstrated in humans with donepezil^17, 54^ and galantamine^55^—two cholinesterase inhibitors that increase acetylcholine in the synaptic cleft and prescribed for the symptomatic treatment of patients with AD. Further research to develop a nicotinic cholinergic index would also be an important tool in drug development as nicotinic reversal has also been successfully reported^56^, therefore an index for nicotinic antagonists could provide a useful non-invasive method to monitor the effects of an important class of drugs.

Furthermore, while improvement of cognitive functions is difficult to quantify in healthy subjects^46, 47^, administration of the agonists may induce changes in the mAChR index that might not be quantifiable with other cognitive tests without the use of a pharmacologic challenge test^57^. Therefore, a more accurate measure of the EEG effects of scopolamine and of cholinergic compounds may result in superior detection of pharmacological (scopolamine reversing) effects. This is very important for drug development, both in terms of proof-of-pharmacology and dose finding. Showing reversal of scopolamine effects by cholinergic compounds (even those proven to be effective in the clinic) is difficult, but this method holds potential: it improves detection of muscarinic anticholinergic EEG effects, so we can expect it to be beneficial at showing the reversal of those effects as well. Moreover, this method may also help to detect cholinergic effects in healthy subjects (or AD patients) who have not been given the scopolamine challenge. The trial data analyzed in this study were recorded during eyes-closed rest; however, the eyes-open rest condition is also used frequently in the neuroimaging literature and future studies should address if the current indices apply equally well to the eyes-open rest EEG.

In conclusion, scopolamine effects on the EEG are clearly present and the spectral ones are well known; however, the mAChR index also accommodates the temporal dynamics to provide deeper insight into the brain’s cholinergic electrophysiology. The index serves as a sensitive biomarker to detect the effect of scopolamine in a dose-dependent manner as well as provide evidence for drug penetration and, therefore, holds potential for being used in experimental pharmacology.

## Methods

### Subjects

Data were obtained from four separate trials conducted at the Centre of Human Drug Research (Leiden, the Netherlands) and approved by a medical ethics committee (Medical Ethics Review Committee of the Leiden University Medical Center, Medical Ethics Review Committee of the Stichting Beoordeling Ethiek Biomedisch Onderzoek, Medical Ethics Review Committee of the Vrije Universiteit Medisch Centrum). All subjects signed a written informed consent prior to participation in the study and were medically screened. All methods were performed in accordance with the relevant guidelines and regulations.

Trial 1 and 2 (P05.168, P05.16) evaluated the effect of investigational glycinergic compounds during a cognitive impairment scopolamine challenge test. A detailed description of the neurophysiologic tests has been reported previously^19, 20^. In the two trials, a total of 83 male healthy subjects aged 18–55 years were recruited. Scopolamine (0.5 mg) or placebo was administered as a 15-minute intravenous infusion. Only the data where subjects received placebo or scopolamine (alone) was used in the analysis. Study periods were separated by a washout period of at least 1 week. The sampling and measurement schedules for the scopolamine challenges were identical for both studies. The measurements were performed during 36 hours treatments periods with 11 measurement time-points from baseline (pre-dose) to 8.5 hrs after scopolamine (or placebo) administration.

Trial 3 (NL38837.056.11) evaluated the effect of a novel α7 nicotinic acetylcholine receptor agonist (α7nAChR) during a scopolamine challenge test. The study recruited a total of 35 subjects, between 65 and 85 years (mixed male and female participants, exact ratio is not known). All subjects received 0.3 mg scopolamine (IV) in 15 minutes. Neurophysiological tests were measured with 8 measurement times from twice at baseline (-1 day) to 6 h after scopolamine administration (open-label). A detailed description of the neurophysiologic tests can be found elsewhere^12, 21^.

Trial 4 (NL33145.029.10) consisted of 40 mild to moderate AD patients aged between 50 and 80 years (mixed male and female patients, exact ratio is not known). The patients were recently diagnosed with “probable AD” (according to NINCDS-ADRDA), had mild to moderate severity of dementia (according to Clinical Dementia Rating Score, CDR of 0.5–2) and scored 18–26 on the Mini-Mental State Examination. Here, we used eyes-closed rest EEG recordings obtained in the baseline before the administration of an investigational drug.

### EEG recordings and pre-processing

EEG recordings were made using silver chloride electrodes fixed at Fz, Cz, Pz, and Oz positions, with the same common reference electrode as for the eye movement registration (according to the international 10/20 system). Electrode resistances were kept below 5 kΩ. EEG signals were obtained from leads Fz-Cz and Pz-Oz and a separate channel to record eye movements (for artefacts). The signals were amplified by use of a Grass telefector (F-15EB/B1) and a 15LT series Amplifier Systems (Grass-Telefactor) with a time constant of 0.3 s and a low-pass filter at 100 Hz. The duration of the recordings was 64 seconds^58^. Sampling frequency was 64768 Hz, afterwards down-sampled to 1012 Hz for the analysis. The ongoing EEG was visually inspected in windows of 10 seconds and sharp transient artefacts were cut out, as well as eye movement and muscle artefacts. Noisy channels were excluded from the subsequent analysis.

### EEG analysis

For the EEG analysis, the Neurophysiological Biomarker Toolbox (NBT) (http://www.nbtwiki.net/)^59^ was used to calculate the biomarkers and custom made scripts were integrated with the NBT analysis pipeline for advanced statistics, employing data mining algorithms to combine the information from multiple biomarkers. We employed biomarker algorithms in order to extract both temporal and spectral information from the EEG signals in the classical frequency bands: delta (1–4 Hz), theta (4–8 Hz), alpha (8–13 Hz), and beta (13–30 Hz). The power in these frequency bands was computed using the Welch method with a 4096-point Hamming window and a frequency resolution of 0.25 Hz. The relative power was calculated by dividing the absolute power in each frequency band with the integrated power in the range 1–45 Hz. The central frequency, *f*_*c*_, and bandwidth, *f*_*σ*_^60^, were computed according to these formulas:

1 $${f}_{c}=\frac{{\sum }_{f={f}_{L}}^{{f}_{H}}fP(f)}{{\sum }_{f={f}_{L}}^{{f}_{H}}P(f)},$$

2 $${f}_{\sigma }=\sqrt{\frac{{\sum }_{f={f}_{L}}^{{f}_{H}}{(f-{f}_{c})}^{2}P(f)}{{\sum }_{f={f}_{L}}^{{f}_{H}}P(f)}},$$

where *f*_*L*_ and *f*_*H*_ represent the lowest and highest frequency that defines a given frequency band, and *P*(*H*) denotes the power at frequency *f*. Thus, the central frequency biomarker provides information about where the power is concentrated in a given frequency band, whereas the bandwidth provides information about how much the power is spread out around the central frequency.

The amplitude envelope was extracted using the Hilbert transform and analyzed for long-range temporal correlations of the power-law form using detrended fluctuation analysis (DFA)^36, 59, 61^. If a sequence of events has a non-random temporal structure with slowly decaying autocorrelations, DFA can quantify how slowly these correlations decay as indexed by the DFA power-law exponent. Signals were filtered using a FIR filter with a Hamming window with a length corresponding to two *f*_1_ Hz cycles for frequency band [*f*_1_, *f*_2_]. To minimize temporal correlations introduced by the FIR filter, DFA was fitted in the interval from 4 to 20 seconds for delta and theta band, from 2 to 20 seconds for alpha and 1 to 20 seconds for the beta band. The oscillation burst lifetime was used to quantify differences in amplitude dynamics of oscillations on short to intermediate time scales (<1 s)^30, 62^. We used a threshold at the median of the amplitude envelope and defined the beginning and the end of an oscillation burst as the time points of crossing this threshold. The duration of oscillation bursts was calculated by taking the 95^th^ percentile of all durations measured within each channel, which we refer to as the “oscillation burst lifetime” biomarker. In total, 20 biomarkers were extracted from each EEG signal. Each of the biomarkers was computed over two bipolar channels (Fz-Cz and Pz-Oz) resulting in a total of 40 features for classification analysis.

### Statistical analysis

Machine learning techniques were used to find the biomarkers that best distinguished the peak effect of scopolamine from the baseline recording or that best distinguished AD patients from healthy controls. From time-dependent curves of EEG biomarkers (Fig. 2), we evaluated 1.5 h after administration of scopolamine as the peak for most EEG biomarkers—in agreement with the peak drug effect (T_MAX_) time point according to the cognitive measurements^12, 21^; therefore, we performed classification on the EEG recorded at baseline and 1.5 h after administration of scopolamine for the development of the mAChR index. For the AD index, we used pre-intervention baseline recordings of AD patients and healthy controls.

A feature matrix was built from the EEG biomarkers—in the form #features × #samples—with the aim of identifying sets of biomarkers that were more discriminative between the two groups than each individual biomarker. Feature selection and classification were performed via the classical machine learning procedure steps: training and testing. In the training phase, the index was developed by applying the feature-selection algorithm to training data and in the test phase, the index was applied to predict the class membership on the test data. The features used for machine learning were z-scored EEG biomarker values. To avoid introducing future information into the classifier, we normalized both the training and the test data by subtracting the mean and dividing by the standard deviation of biomarker values from the training data only.

Indices were identified by applying the classification algorithm to the whole dataset (Trials 1 and 2 for mAChR; Trials 3 and 4 for the AD index); however, cross-validation was used to evaluate the stability of the result, i.e., classification with 100 different splits of the data into training and test sets were performed to obtain the median and median absolute deviation of the classification performance, which provides an estimate of the classification performance on an “unknown” sample^63^. To this end, we used the cross-validation with 70/30% random splitting, i.e., from a random permutation of the subjects, 70% were used for training and 30% for testing. The training set consisted of 115 EEG recordings, tested on 48 recordings for the mAChR index (Trial 1 and 2). The total number of recordings is twice the number of subjects: per subject, the baseline EEG recording was used as the first sample and the peak drug effect recording as the second sample. For the AD index, the training and test set consisted of 53 and 22 recordings (and subjects), respectively (Trial 3 and 4). Cross-validation is not a computationally expensive operation, taking about 10 minutes on a modern desktop computer (for 100 cross-validations, using parallel computing in MATLAB).

### Elastic net logistic regression

Because of correlation between some of the features and an interest in reducing the number of features, we chose to use the elastic net^64^, which has sparsity and grouping of correlated features as properties. Additionally, elastic net is an embedded method, which performs both feature selection and classification. It is a regularized logistic regression that bridges the gap between lasso^65^ and ridge regression^66^ by combining their penalties and optimizing the number of features included in the integrated index through minimizing the function:

3 $$L({\lambda }_{1},{\lambda }_{2},\beta )=|y-X\beta {|}^{2}+{\lambda }_{1}||\beta |{|}_{1}+{\lambda }_{2}||\beta |{{|}_{2}}^{2},$$

where *X* is the feature matrix, *y* is the response vector (the labels) *β* the weights, and *λ*_1_ and *λ*_2_ coefficients determining the influence of the *L*_1_ and *L*_2_ norm penalties, respectively. The first term is similar to logistic regression while the second and third terms form the elastic net penalty function. If we denote: *α* = *λ*_2_/(*λ*_1_ + *λ*_2_), then the elastic net penalty can be rewritten as $(1-\alpha )||\beta |{|}_{1}+\alpha ||\beta |{{|}_{2}}^{2},$ where *α* acts as the balancing term between the *L*_1_ and *L*_2_ norm penalties. We optimized *α* in a 5-fold cross-validation procedure and found the best classification performance with *α* = 0.5 (results not shown).

By minimizing the *L*-function, we obtain the set of *n* selected features corresponding to the ones with highest *β* values. If *p* is the probability that an EEG recording belongs to the peak scopolamine condition, then the odds ratio is p/(1 − p), which is the ratio of the probability of peak scopolamine to the probability of baseline recording. Logistic regression models the log odds ratio as a linear combination of the independent variables, via this equation:

4 $$\mathrm{ln}(\frac{p}{1-p})={\beta }_{0}+{\beta }_{1}\,{f}_{1}+\cdots +{\beta }_{n}\,{f}_{n},$$

where f_i_ are the features and *β*_*i*_ the associated weights. The log odds can be transformed back to probabilities as:

5 $$p(t)=\frac{1}{1+\exp (-t)},t={\beta }_{0}+{\beta }_{1}\,{f}_{1}+\cdots +{\beta }_{n}\,{f}_{n}.$$

The size of the final set of selected features is estimated as the one that gives the maximum classification performance on the training set, while keeping the feature set as small as possible. To obtain this set, we compared the accuracy of classifiers using the *k* highest *β*-s, with *k* ranging from 1 to the number of features *n* and selected the smallest feature set with optimal performance outcome measures.

### Classification outcome evaluation

Elastic net logistic regression algorithm was used for developing two integrated indices: 1) The mAChR index, which is classifying whether an EEG was recorded during the baseline or when scopolamine has been administrated; 2) The AD index, which is classifying whether an EEG was recorded from a healthy elderly or an AD patient. To evaluate the classification performance of the indices we used four different measures. In the case of the mAChR index, they are defined as:

Accuracy (AC): (number of correctly classified scopolamine and baseline recordings)/(total number of recordings).

Sensitivity (SE): (number of correctly classified scopolamine recordings)/(number of scopolamine recordings).

Specificity (SP): (number of correctly classified baseline recordings)/(number of baseline recordings).

Precision (PR): (number of correctly classified scopolamine recordings)/(number of recordings classified as scopolamine).

Area Under Curve (AUC): area under the Receiver Operating Characteristic (ROC) curve, which plots the true positive rate (SE) against the true negative rate (1-SP) as the discrimination threshold of the classifier is varied. A higher AUC means better classification performance.

Analogous definitions apply for the classification performance of the AD index.
